# *Helicobacter pylori* Induces miR-155 in T Cells in a cAMP-Foxp3-Dependent Manner

**DOI:** 10.1371/journal.pone.0009500

**Published:** 2010-03-02

**Authors:** Lina Fassi Fehri, Manuel Koch, Elena Belogolova, Hany Khalil, Christian Bolz, Behnam Kalali, Hans J. Mollenkopf, Macarena Beigier-Bompadre, Alexander Karlas, Thomas Schneider, Yuri Churin, Markus Gerhard, Thomas F. Meyer

**Affiliations:** 1 Department of Molecular Biology, Max Planck Institute for Infection Biology, Berlin, Germany; 2 Microarray Core Facility, Max Planck Institute for Infection Biology, Berlin, Germany; 3 Laboratory of Gastroenterology III, Department of Medicine II, Technical University of Munich, Munich, Germany; 4 Medical Clinic I, Charité, Berlin, Germany; University of Hyderabad, India

## Abstract

Amongst the most severe clinical outcomes of life-long infections with *Helicobacter pylori* is the development of peptic ulcers and gastric adenocarcinoma - diseases often associated with an increase of regulatory T cells. Understanding *H. pylori*-driven regulation of T cells is therefore of crucial clinical importance. Several studies have defined mammalian microRNAs as key regulators of the immune system and of carcinogenic processes. Hence, we aimed here to identify *H. pylori-*regulated miRNAs, mainly in human T cells. MicroRNA profiling of non-infected and infected human T cells revealed *H. pylori* infection triggers miR-155 expression *in vitro* and *in vivo*. By using single and double *H. pylori* mutants and the corresponding purified enzymes, the bacterial vacuolating toxin A (VacA) and γ-glutamyl transpeptidase (GGT) plus lipopolysaccharide (LPS) tested positive for their ability to regulate miR-155 and Foxp3 expression in human lymphocytes; the latter being considered as the master regulator and marker of regulatory T cells. RNAi-mediated knockdown (KD) of the Foxp3 transcription factor in T cells abolished miR-155 expression. Using adenylate cyclase inhibitors, the miR-155 induction cascade was shown to be dependent on the second messenger cyclic adenosine monophosphate (cAMP). Furthermore, we found that miR-155 directly targets the protein kinase A inhibitor α (PKIα) mRNA in its 3′UTR, indicative of a positive feedback mechanism on the cAMP pathway. Taken together, our study describes, in the context of an *H. pylori* infection, a direct link between Foxp3 and miR-155 in human T cells and highlights the significance of cAMP in this miR-155 induction cascade.

## Introduction

*Helicobacter pylori* is a Gram-negative microaerophilic bacterium estimated to colonize more than half the world's population. It typically establishes a life-long infection within the stomach, where it induces a marked immune response with local infiltration of neutrophils, lymphocytes and macrophages. *H. pylori* is the main cause of peptic ulceration (15% of infections) and gastric adenocarcinoma (0.5 to 2% infections) (for review, see [1] and it has been shown to cause B cell mucosa associated lymphoid tissue (MALT) lymphoma [2]. While several mechanisms involved in adenocarcinoma induction have been described, the mechanisms underlying the onset of other *H. pylori*-implicated lymphomas remain obscure.

MicroRNAs (miRNAs) are non-protein coding ∼22 nucleotide RNAs that induce translational repression and/or degradation of their mRNA targets [3], [4]. Many miRNAs are differentially regulated in cancer, for example, miR-34a is involved in p53-mediated apoptosis in pancreatic cancer [4]–[6]; in primary breast cancer, nine miRNAs were up-regulated, including miR-21, miR-181b and miR-155 [7]. The implication of miRNAs in cancer development is particularly true for the let-7 family members. Convergence of abundant *in vitro* and *in vivo* data has identified these miRNAs as tumor-suppressors that regulate multiple oncogenes such as Ras, Myc or HMGA-2 (reviewed in [8]).

A growing body of evidence suggests bacterial and viral infection of mammalian and plant cells can modulate miRNA expression [9]. In mammalian cells, the Epstein-Barr virus (EBV) has been shown to trigger miR-21, miR-155 and miR-146a expression [10], which might play a role in the development of EBV associated Burkitt's lymphoma. Treatment of immune cells with bacterial lipopolysaccharide (LPS; *Salmonella* and *Escherichia coli)* led to the induction of miR-155, miR-132 and miR-146a expression [11]. Other studies have reported the induction of miR-155 expression in murine primary macrophages by a range of toll-like receptor ligands [12] and that LPS stimulation can also lead to the down-regulation of miRNAs such as miR-125b, as demonstrated in murine macrophages and C57Bl6 mice [11]. Most recently, induction of miR-155 expression upon *H. pylori* infection of gastric epithelial cells and tissues has been reported [13]. T cells are key players *in vivo* in terms of inflammation and B cell lymphoma initiation. To date, however, there has been no study of the regulation of miRNAs by *H. pylori* in T cells.

*H. pylori* harbours several virulence factors (for review, see [1], [14]) that contribute to the genesis of inflammation. Important virulence factors are the cagPAI, encoding a type IV secretion system, the vacuolating cytotoxin (VacA), responsible for vacuolation, apoptosis and inhibition of Interleukin (IL-2) secretion in T cells [15], [16] and the γ-glutamyl transpeptidase (GGT), which inhibits T cell proliferation [17].

Here, we investigated the differential regulation of miRNAs by the carcinogenic bacterium *H. pylori* mainly in T cells, and the underlying bacterial and host mechanisms associated with their regulation during the course of an infection. Our results revealed that miR-155 is up-regulated upon *H. pylori* infection *in vitro* and *in vivo,* and its activation is under control of the Foxp3 transcription factor in T cells. We also identified bacterial factors that mediate miR-155 expression. Furthermore, we provide evidence that intracellular cyclic adenosine monophosphate (cAMP) is a key component of this bacterial activation and that miR-155 targets protein kinase A inhibitor α (PKIα) in order to further increase cAMP production.

## Results

### *H. pylori* Induces Up-Regulation of miR-155 Expression in Different Cell Lines

To examine whether *H. pylori* regulates miRNA expression in T cells, a microarray analysis of 248 mature miRNAs of human and murine origin was conducted with the human T cell line Jurkat. Regulation of miRNAs has been previously reported in epithelial cell lines [13], [18], therefore we sought to substantiate these findings in two different human epithelial cell lines (AGS and MKN74). We also compared the regulation response within a murine macrophage cell line (J774a). The cells were infected for 3 hours with two different strains of *H. pylori* (P12 and Hp76) depending on their human or murine origin, respectively. In both epithelial cell lines (AGS and MKN74), a mild up-regulation (∼2-fold) of miR-26a was observed. In the macrophage cell line J774a, murine miR-292-5p, miR-298 and miR-290 were up-regulated 2.7-, 2.2- and 2-fold, respectively. In Jurkat cells, only miR-155 was up-regulated (3.3-fold). Up-regulation of miR-155 was approximately 3-fold in J774a and MKN74 cells, and 7.5-fold in AGS cells (microarray deposited data, GSE18616). Since miR-155 was the only common miRNA significantly induced by *H. pylori* in all tested cell lines, it was selected for further analysis.

**Figure 1:**
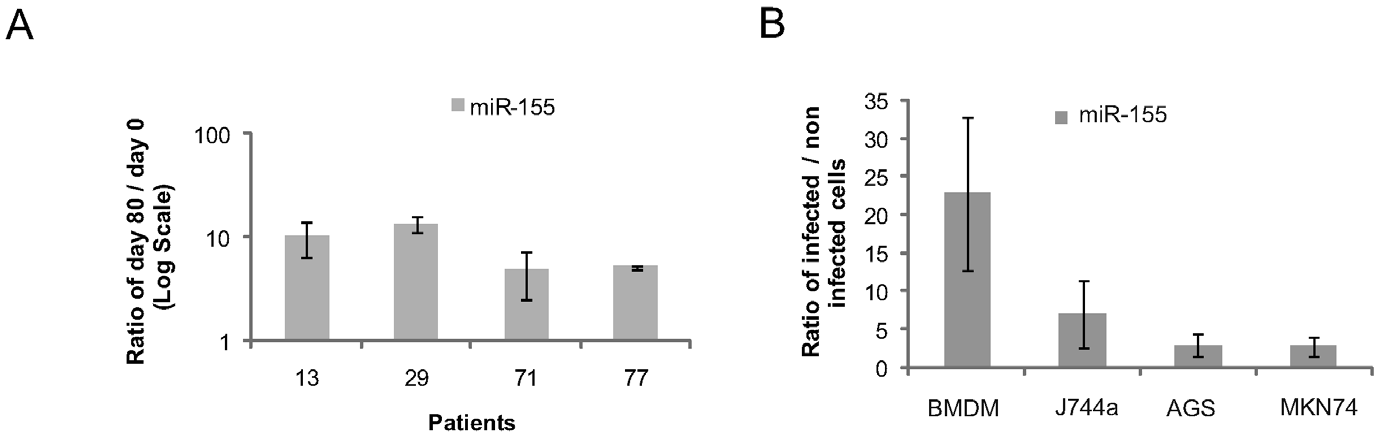
*H. pylori* induces miR-155 expression *in vivo* and *in vitro*. (A) *In vivo* analysis of miR-155 expression in human gastric biopsies. Total RNA extracted from the antrum of four patients before and after 80 days of infection (patient 13, 29, 71 and 77) and analysed by qPCR was used to show the induction of miR-155 expression by *H. pylori* in the gastric mucosa. (B) miR-155 expression in primary murine macrophages (Bone Marrow Derived Macrophages), J774a, AGS and MKN74 cell lines after *H. pylori* infection (MOI 100, 3 h) as monitored by qPCR. Murine cells (BMDM and J774a) were infected with Hp76 strain while human cells (AGS and MKN74) were infected with the P12 strain. Results are expressed as a ratio between infected and non-infected samples. Error bars show standard deviation (SD) of three independent experiments.

Next, we measured miR-155 expression *in vivo*, using human gastric biopsies. Four patients, initially negative for *H. pylori* infection ( =  day 0), were challenged with a *H. pylori* strain lacking a functional cagPAI [19], [20]. Eighty days post-infection (p.i.) (day 80), biopsies containing a mixture of immune and non-immune cells were taken from the antrum of the volunteers and RNA was extracted. The relative numbers of cell types were quantified by real time RT-PCR detection of specific cellular markers such as cytokeratin 18 (epithelial cells), CD3 (T lymphocytes) and CD11c (macrophages and dendritic cells). Numbers of epithelial or T cells did not change substantially during infection with *H. pylori* over 80 days; however, macrophages and/or dendritic cells were strongly recruited to the site of inflammation (up to 16-fold increase; Figure S1). Comparison of miR-155 expression levels in each patient before (day 0) and after infection (Day 80) revealed a strong up-regulation (up to ∼13-fold) of miR-155 (Fig. 1A), indicating that cagPAI is unnecessary for miR-155 induction.

**Figure 2:**
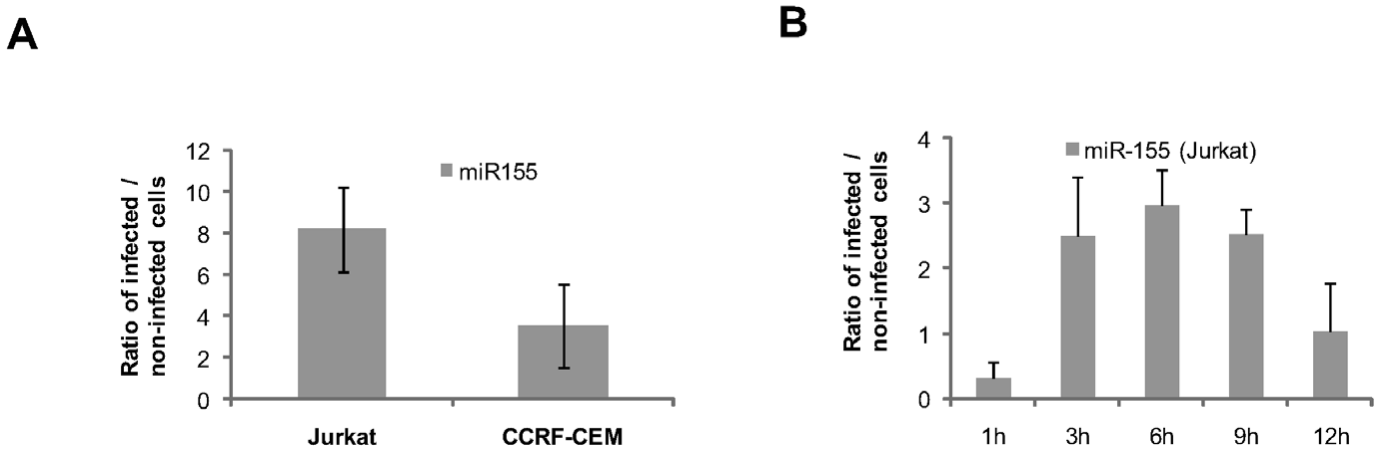
*H. pylori* induces miR-155 expression *in vitro*. (A) Results of qPCR of miR-155 expression in Jurkat and CCRF-CEM cell lines after *H. pylori* infection (MOI 100, 3 h). Data are mean ±SD of three independent experiments. (B) Kinetics of miR-155 expression upon *H. pylori* infection in Jurkat cells. Total RNA was extracted from cells infected with the P12 strain and non-infected cells at different time points (1 h, 3 h, 6 h, 9 h,12 h) and assayed for miR-155 by qPCR. Results are expressed as a ratio between infected and non-infected samples. Results are normalized to expression levels of the small RNA U6 (RNU6B), which remains unchanged by the bacterial stimulus. Error bars show SD of at least three independent experiments.

To further validate our observations, infections with the wild type *H. pylori* strains P12 and Hp76 were performed in the same four cell lines, plus primary murine derived macrophages (BMDM) isolated from C57Bl6 mice. Quantitative-PCR (qPCR) analysis of miR-155 expression confirmed our microarray data (Fig. 1B), even when a lower multiplicity of infection (MOI) was used (MOI 10 and 50; data not shown). Northern blotting and qPCR revealed a clear up-regulation of miR-155 by *H. pylori* in J774a cells (data not shown). Since miR-155 is expressed at low levels in T cells [21], it was not detectable using Northern blotting. Instead, we used qPCR to confirm miR-155 induction in Jurkat cells and another T cell line, CCRF-CEM (Fig. 2A). Expression kinetics of miR-155 in Jurkat cells were monitored over a 12 h infection time course, revealing a sharp up-regulation of miR-155 expression at 3 h p.i., which continued to increase until 6 h p.i., before gradually declining to levels comparable to non-infected cells by 12 h p.i. (Fig. 2B).

### miR-155 Induction Is *H. pylori* LPS Dependent

**Figure 3:**
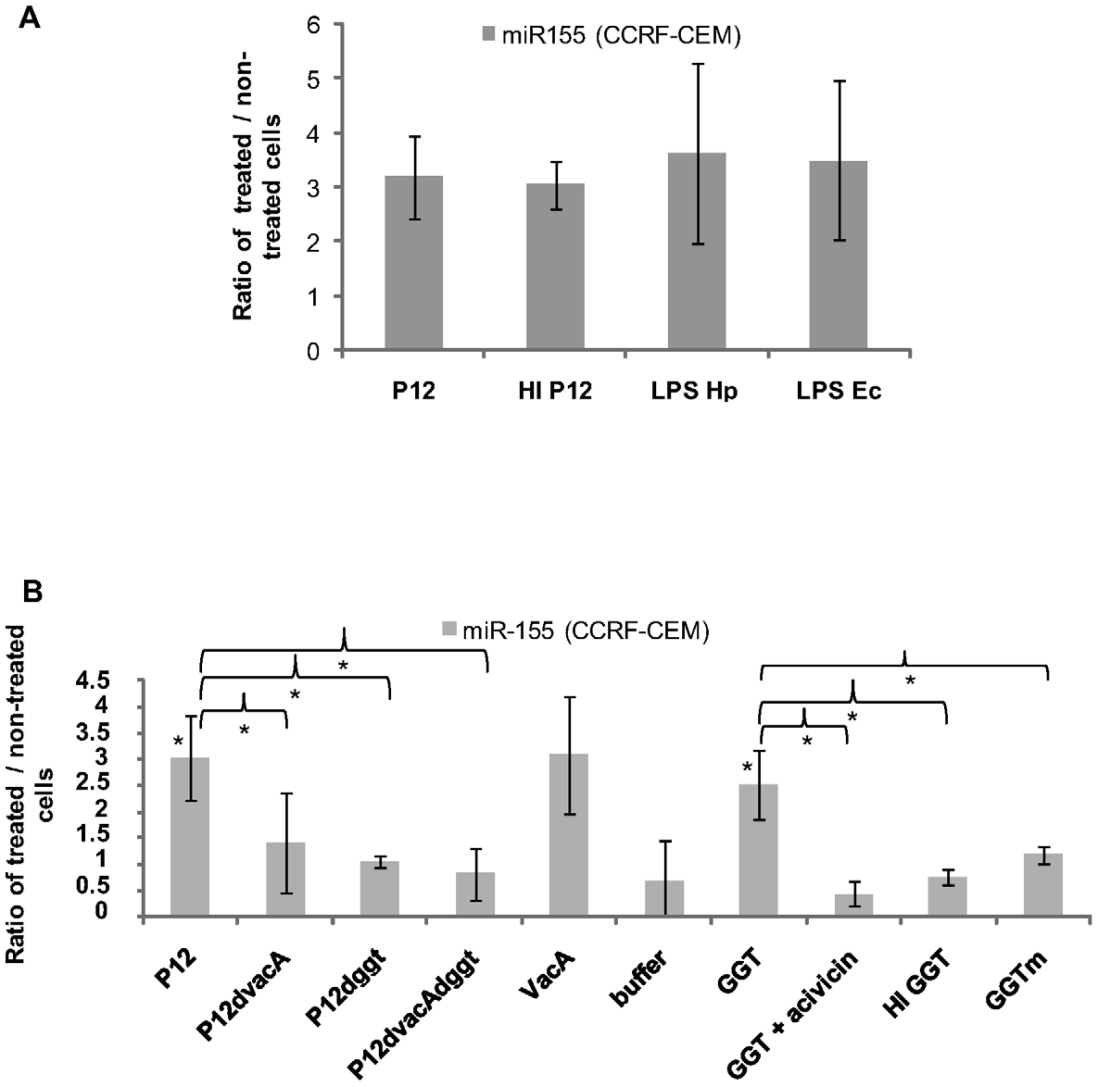
*H. pylori* induces miR-155 expression in an LPS, VacA- and GGT-dependent manner. (A) CCRF-CEM were co-cultured with live or heat inactivated (HI) *H. pylori* (P12, MOI 100, 3 hours) or incubated with purified *E.coli* and *H. pylori* LPS (100 and 500 ng/ml). miR-155 expression was then measured by qPCR. Results are normalized to expression levels of RNU6B. (B) CCRF-CEM cells were infected with the wild type strain P12, the single mutants (P12d*vacA* and P12d*ggt*) and the double mutant (P12d*vacAdggt*) at an MOI of 100 for 3 h. The T cells were also incubated with the purified enzyme VacA and its buffer. The GGT enzyme was tested for miR-155 expression with or without the GGT inhibitor (acivicin). Acivicin treatment alone did not affect miR-155 expression. A mutant form of the GGT (GGTm) and the heat-inactivated protein (HI GGT) were negative for miR-155 induction. Results in (A) and (B) are expressed as a ratio between infected and non-infected samples. Error bars show SD of at least two independent experiments. * p value≤0.05.

Since miR-155 induction can occur after LPS treatment of macrophages [11], [12], we tested whether *H. pylori* LPS regulates miR-155 expression. CCRF-CEM cells were incubated with purified *H. pylori* LPS (500 ng/ml) and miR-155 expression was assessed by qPCR. *E.coli* LPS (100 ng/ml) and whole live *H. pylori* (P12) were used as positive controls. Heat-inactivated *H. pylori* (HI P12) was used to test whether miR-155 induction occurs via a passive or an active mechanism. All stimuli induced miR-155 expression to a similar extent (∼3-fold increase) (Fig. 3A), suggesting that miR-155 induction by *H. pylori* is partially LPS dependent.

### Induction of miR-155 Expression Is VacA and GGT Dependent

The mouse-adapted strain Hp76 induces miR-155 up-regulation in macrophages (see Fig. 1B). Since this strain does not possess a functional cagPAI (Figure S2), we concluded that cagPAI is not necessarily required for induction of mir-155. To identify the bacterial factors that could be responsible for miR-155 induction in T cells, we investigated the role of two key virulence proteins, VacA and GGT, known to affect T cell function in terms of IL-2 secretion and proliferation [15], [17] (Fig. 3B). Expression of miR-155 was measured by qPCR after infection of CCRF-CEM cells with either single mutants of *H. pylori* (P12Δ*vacA*, P12Δ*ggt*) or the double mutant (P12Δ*vacA*Δ*ggt*). In all mutants tested, miR-155 induction was decreased in comparison to the wild type *H. pylori*. In mutants lacking GGT, this decrease was most pronounced (∼3-fold) (Fig. 3B). However, when cells were incubated with the purified VacA and GGT proteins, miR-155 expression increased 2.5 to 3-fold (Fig. 3B). Protein concentrations used were similar to those previously reported for the inhibition of IL-2 secretion and proliferation [15], [17]. Since the GGT protein was obtained as a recombinant enzyme from *E. coli* and hence was possibly contaminated by bacterial LPS, we co-incubated cells with the GGT inhibitor acivicin, prior to GGT treatment. Consequently, miR-155 induction was abolished (Fig. 3B). Acivicin treatment did not affect cell viability at the used concentration, as confirmed by the WST1 viability assay (Figure S3). Heat inactivation of GGT (HI GGT) or the use of a mutant lacking catalytic activity (GGTm) demonstrated that only the active catalytic form of GGT can induce miR-155 expression. These results indicate that miR-155 induction depends on multiple bacterial factors, some of which are already known as key players in *H. pylori* virulence.

### miR-155 Expression Is *Foxp3-*Dependent

**Figure 4:**
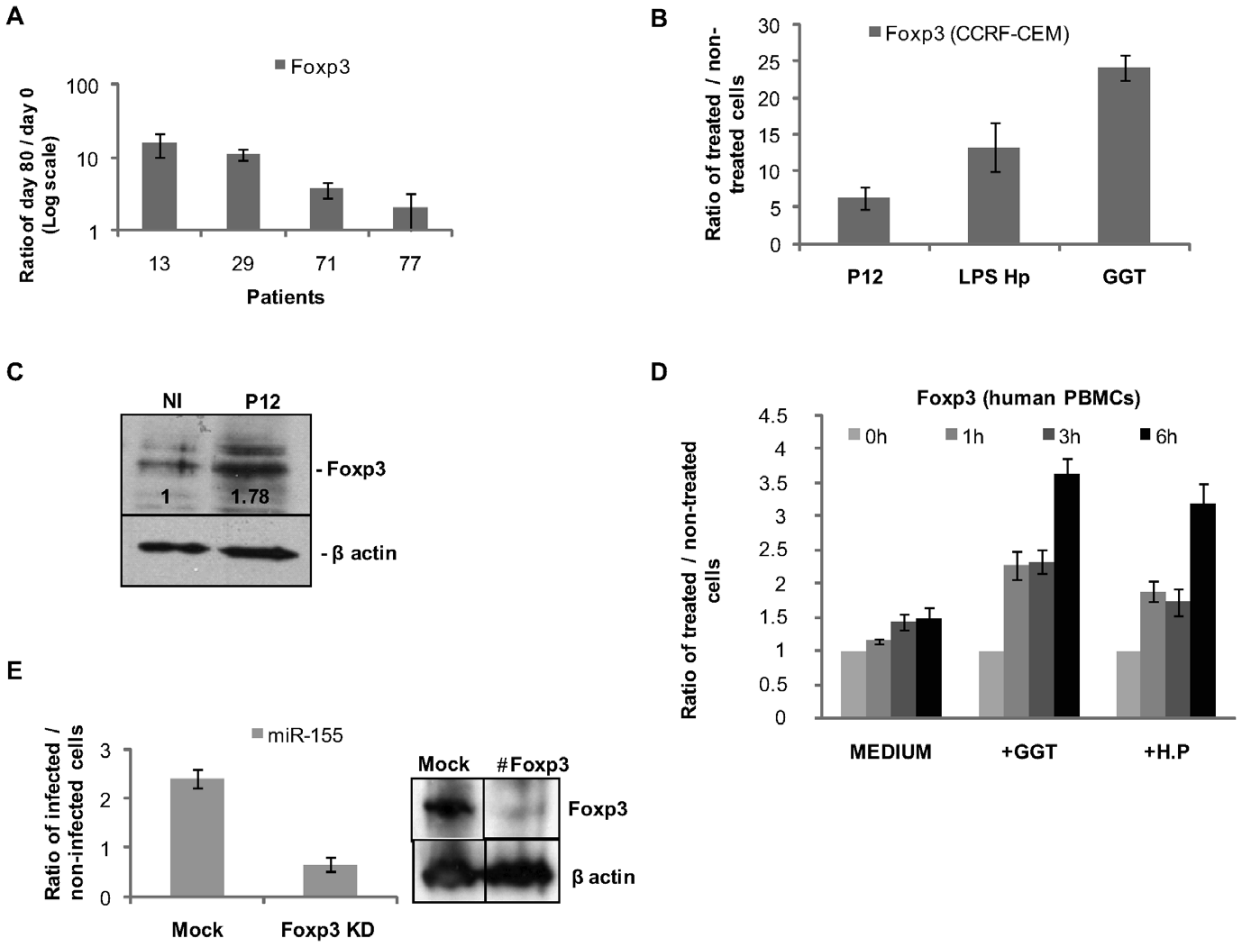
miR-155 expression in T cells is dependent on the *H. pylori*-mediated induction of Foxp3. (A) *In vivo* analysis of Foxp3 expression in human gastric biopsies. Total RNA extracted from the antrum of four patients before and after 80 days of infection (patient 13, 29, 71 and 77) was used for the measurement of Foxp3 expression by qPCR. (B) Total RNA from CCRF-CEM cells was assayed for Foxp3 mRNA expression after *H. pylori* infection or incubation with *H. pylori* LPS and GGT, and compared to non-infected cells. qPCR data show an induction of Foxp3 after challenge with live bacteria, LPS and purified GGT. Data are mean ±SD of two independent experiments. (C) Western blot analysis of Foxp3 protein induction in CCRF-CEM cells infected with *H. pylori* (P12, 3 h). Actin serves as a loading control. Foxp3 band intensities shown. Blots are representative of two independent experiments. (D) Human PBMCs were assayed by qPCR for Foxp3 expression after *H. pylori* infection or GGT treatment over time (1 to 6 h). The results obtained from 5 individual donors show an increase of Foxp3 mRNA after 6 h. (E) miR-155 expression was measured by qPCR in the Foxp3 KD CCRF-CEM cells at 3 h p.i. Results are expressed as a ratio between infected and non-infected samples. Error bars show SD of two independent sets of experiments. Foxp3 (#Foxp3) KD was achieved using siRNA technology and confirmed by Western blot 48 hours post-transfection.

Next, we investigated the role of host cell factors in the up-regulation of miR-155. Since the transcription factor Foxp3 is reported to bind the miR-155 promoter in primary murine CD4^+^ CD25^+^T cells [22], we speculated Foxp3 may regulate miR-155 expression. First we analysed Foxp3 expression *in vivo*, using biopsies from the four human volunteers. Both Foxp3 and miR-155 expression were up-regulated by up to ∼13-fold (Fig. 4A), indicating that *H. pylori* is able to trigger Foxp3 expression.

Upon infection of CCRF-CEM cells with *H. pylori*, *H. pylori* LPS or GGT, Foxp3 mRNA levels were increased (∼6.4-, ∼13- and ∼24-fold increase, respectively; Fig. 4B). At the protein level, *H. pylori* elicited a 1.7-fold up-regulation of Foxp3 at 3 h p.i. (Fig. 4C). We validated these results using primary cells (PBMCs) from five human donors negative for any previous *H. pylori* infection (Fig. 4D). Foxp3 mRNA induction was observed 6 h after *H. pylori* infection or GGT treatment in all donors. To demonstrate that Foxp3 is directly involved in miR-155 induction, we induced a transient knockdown (KD) of Foxp3 (#Foxp3) in non-infected and P12-infected CCRF-CEM cells using RNA interference (∼80% KD). No up-regulation of miR-155 was detected (Fig. 4E), indicating that Foxp3 KD abolished expression of this miRNA in CCRF-CEM cells. Similar data were obtained for Jurkat cells (data not shown). Taken together, these data indicate a strong correlation between Foxp3 and miR-155 expression *in vivo* and *in vitro*.

### cAMP Pathway Dependent Regulation of Foxp3 and miR-155 Expression

**Figure 5:**
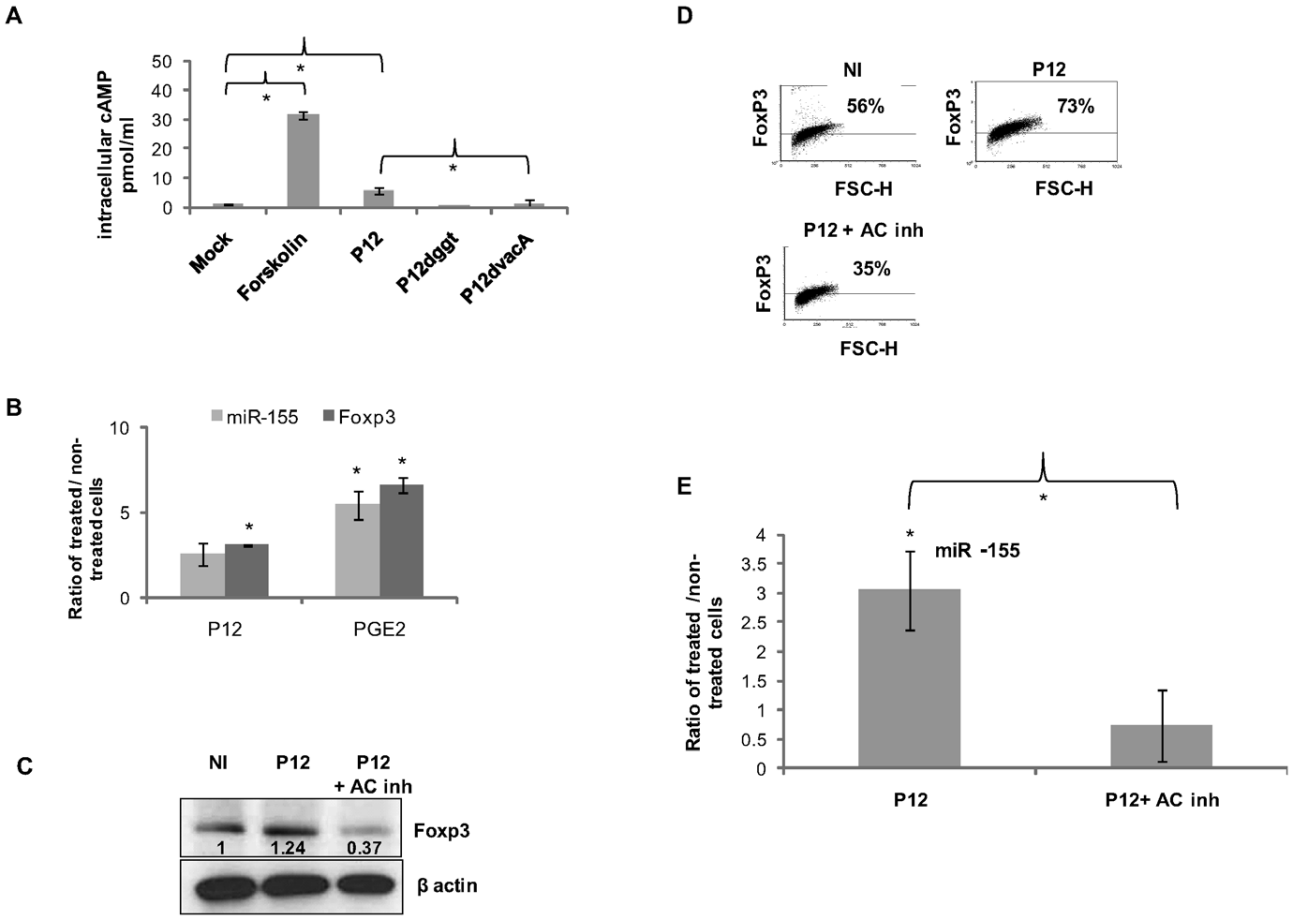
Foxp3 and miR-155 expression are mediated by cAMP induction in T cells. (A) Intracellular levels of cAMP were measured in Jurkat cells after 30 min of forskolin treatment (positive control) or *H. pylori* infection (wild type strain P12 and the mutants d*ggt* and d*vacA*). (B) Activation of ACs induces Foxp3 and miR-155 expression in T cells. Jurkat cells were incubated for 3 h with the wild type strain of *H. pylori* (P12) or with the AC agonist PGE2 (26 µM). Foxp3 and miR-155 expression were measured by qPCR. Results are expressed as a ratio between treated and/or infected, and mock samples. One hour prior to P12 infection, Jurkat cells were incubated with 50 µM of the irreversible AC inhibitor MDL-12,330A (AC inh). Foxp3 expression was then assessed by Western blot (C) and FACS (D) and compared to non-infected and P12 infected cells. Foxp3 band intensities are shown in (C). Results depicted in (C) and (D) are representative of two independent experiments. (E) miR-155 levels were measured in *H. pylori* infected CCRF-CEM cells with or without the AC inhibitor (AC inh) by qPCR. Data are mean ±SD of at least four independent experiments. * p value≤0.05.

Foxp3 expression is thought to be regulated by the CREB transcription factor [23]. Since CREB is phosphorylated by Protein Kinase A (PKA), which is activated by cAMP [24], we measured intracellular cAMP levels in T cells (Jurkat) after 30 min of infection with wild type and mutant *H. pylori* strains (Fig. 5A). The adenylate cyclase (AC) agonist, forskolin, served as a positive control. Intracellular cAMP levels were drastically increased after infection with the wild type *H. pylori*, in comparison to the mutant strains. Absence of VacA protein and GGT enzyme prevented, or at least severely restricted, induction of cAMP, indicating that the *H. pylori-*induced elevation of cAMP is VacA- and GGT- dependent. Next, we investigated whether cAMP induction is involved in Foxp3 and miR-155 expression by incubating CCRF-CEM cells with the AC agonist PGE2. PGE2 induced an increase in Foxp3 mRNA levels and miR-155 expression after 3 h of incubation; the level of up-regulation after PGE2 addition was comparable to that observed after *H. pylori* infection (Fig. 5B). These results demonstrated that AC is activated, indicating that the cAMP pathway induces Foxp3 and miR-155 expression. To further substantiate our results, T cells were incubated with an irreversible inhibitor of AC, MDL-12330A, 1 h before infection with *H. pylori*. After blockage of the cAMP pathway, Foxp3 protein expression decreased, as shown by Western blot and FACS (Fig. 5C and D). In the presence of the inhibitor, miR-155 expression decreased by 75% after *H. pylori* treatment, as measured by qPCR (Fig. 5E). Thus, our findings show that the cAMP cascade is involved in Foxp3 induction and subsequent miR-155 expression in T cells upon *H. pylori* infection.

### *H. pylori* and miR-155 Induced Over-Expression Down-Regulates PKIα Levels

**Figure 6:**
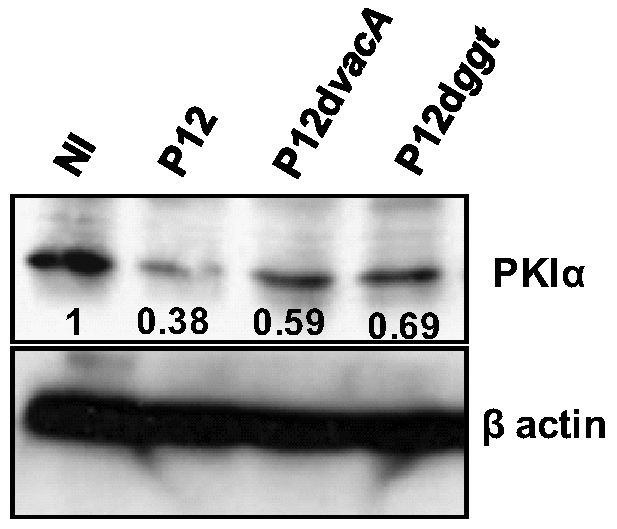
*H. pylori* infection decreases PKIα protein levels. CCRF-CEM cells were infected with *H. pylori* P12 and the mutants lacking VacA (P12d*vacA*) and GGT (P12d*ggt*) proteins and assayed for PKIα protein levels 3 h p.i. PKIα band intensities are shown. Actin serves as a loading control. Blots are representative of two independent experiments.

**Figure 7:**
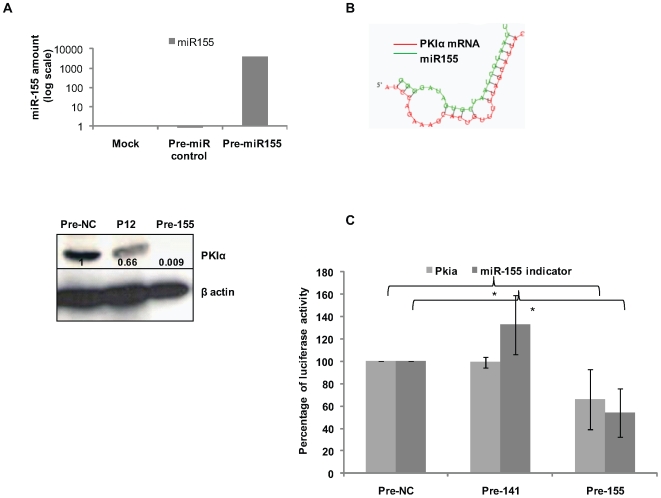
Over-expression of miR-155 abolishes PKIα expression in T cells. (A) T cells were transfected with the precursor of miR-155 (Pre-155) and a negative control (Pre-NC). The over-expression of miR-155, confirmed by qPCR (see graph), induced a dramatic decrease of PKIα protein levels (Band intensities are shown on blot). Results are representative of two independent experiments (B) PicTar prediction of the binding of miR-155 to the 3′UTR of PKIα. (C) 293T cells were transfected with a miR-155 precursor (Pre-155), miR-141 precursor (Pre-141) or the negative control (Pre-NC). Later, the cells were transfected either with the 3′UTR containing plasmid (PKIa UTR), or the miR-155 indicator plasmid (miR-155 indicator). Co-transfection with the *Renilla* plasmid (pRLSV40) was achieved to normalize the data. Luciferase activities were measured. Luciferase activity is shown as a percentage on the y-axis. miR-155 over-expression (checked by the decrease of the luciferase activity of the indicator plasmid) induces a decrease of the firefly luciferase activity of the PKIα plasmid. Data are mean ± SD of two independent experiments. * p value≤0.05.

cAMP mediates a diverse array of cellular responses by activating PKA. Activation of PKA occurs when cAMP binds to the two regulatory subunits of PKA resulting in the release of the two catalytic subunits [25], [26]. We found PKIα protein levels decreased 3 h after *H. pylori* infection of T cells in a GGT- and VacA-dependent manner (Fig. 6). To test if miR-155 was involved in this down-regulation, miR-155 was transiently over-expressed in T cells (∼4250-fold increase of miR-155) (Fig. 7A). Over-expression induced a dramatic decrease of the PKIα protein level showing that the effect is mediated by miR-155 (Fig. 7A). The bioinformatic database PicTar (http://pictar.mdc-berlin.de/) predicts miR-155 targets human PKIα and the core binding sequence has a 7-mer match in the 3′UTR (Fig. 7B). Therefore, we speculated that miR-155 directly targets PKIα. Hence, we performed a luciferase reporter assay after generating a construct in which the firefly luciferase mRNA is fused to the 3′ÚTR of PKIα (PKIa UTR). HEK 293T cells were first co-transfected with the miR-155 precursor or the miR-141 precursor (negative control) or another negative control precursor (NC). At 16 h post transfection, cells were then co-transfected with the *Renilla* luciferase-containing plasmid (pRLSV40) for normalisation and the PKIa UTR plasmid. To assess miR-155 over-expression, an indicator plasmid containing the complementary sequence of miR-155 fused to the firefly luciferase (miR-155 indicator) was simultaneously transfected. Following miR-155 transfection luciferase activities of both the indicator plasmid and the 3′UTR of the PKIα reporter were reduced (∼48% and ∼36%, respectively) in comparison to the negative control precursor, indicating successful miR-155 over-expression (Fig. 7C). Over-expression of miR-141 did not affect luciferase activity encoded by the PKIα reporter. Thus, we conclude PKIα is a direct functional target of miR-155.

## Discussion

While gastric cancer has been strongly linked to the *H. pylori* CagA protein [27], the mechanisms underlying other carcinogenic outcomes, such as MALT lymphoma, are poorly understood. Since miRNAs are involved in the development of a variety of lymphomas [28], we investigated the differential induction of miRNAs after *H. pylori* infection in T cells. Interestingly, our microarray screen identified miR-155, the functional product of an exon within the non-protein coding gene *bic* (B-cell integration cluster) [29], as the only miRNA induced in Jurkat cells. It was the only commonly activated miRNA in different cell lineages 3 h post infection with *H. pylori*. Up-regulation of miR-155 was validated in primary monocytes after stimulation with *H. pylori*, as well as *in vivo* by testing human gastric biopsies from patients that had previously participated in a clinical *H. pylori* challenge study [19].

We investigated the role of key bacterial endotoxins and virulence factors as well as host signalling processes in the expression of miR-155. Consistent with previous findings for macrophages [12] and dendritic cells [30], [31], here we demonstrated that miR-155 expression in lymphocytes is induced by *H. pylori* LPS. We also observed that the *H. pylori-*specific virulence factors VacA and GGT are necessary for the full induction of miR-155. VacA is a secreted pore-forming protein that interferes with several cellular signalling events, including calcium influx and nuclear translocation of NFAT (nuclear factor of activated T cells), thereby preventing T cell activation and proliferation [32]. GGT is also secreted into the extracellular medium where it mediates the extracellular cleavage of glutathione, leading to reactive oxygen species production [33] and induction of a cell cycle arrest in lymphocytes [17]. Although we observed that purified LPS alone was sufficient to induce expression of miR-155, in mutants lacking GGT activity (P12Δ*ggt* and P12Δ*vacA*Δ*ggt*) this expression was completely suppressed. These findings indicate that the miR-155-inducing activity of the LPS is impaired in the GGT mutants and further suggest bacterial GGT activity plays a supportive role in LPS-dependent miR-155 expression. However, comparison of these results is hampered by the necessary usage of different amounts of LPS in both experiments (500 ng/ml purified LPS vs. bacterial LPS in an infection at MOI 100).

To provide a more complete assessment of the mechanisms of miR-155 regulation during *H. pylori* infection, host transcription factors were also investigated. The regulation of miR-155 has recently been shown to be dependent on the activator protein 1 (AP-1) pathway in B cells after B cell receptor activation [34] and in primary murine macrophages [12]; however, as Foxp3 is reported to control miR-155 expression in T cells [35], we focused on the role of this factor. Increased numbers of Foxp3+ T cells have been found in the gastric mucosa of *H. pylori* infected adenocarcinoma patients in comparison to non-infected individuals [36]. Our study showed that *H. pylori* infection induces Foxp3 expression in CD4+ cells. Using a transient Foxp3 KD, we demonstrated that Foxp3 is mandatory for miR-155 expression in T cells. This transcription factor was previously considered as the master regulatory gene in regulatory T cells. By showing that Foxp3 induces miR-155 our study hints at a new role for Foxp3 in B cell lymphoma development.

Previous studies have suggested PGE2 could trigger Foxp3 expression in human T cells [37] and that cAMP is a component of regulatory T cells functions [38]. Here, we showed that Foxp3 and miR-155 are induced by the AC agonist PGE2, and that VacA and GGT could induce expression of miR-155. Both VacA and GGT have been reported to enhance cyclo-oxygenase 2 expression and hence PGE2 secretion in mammalian cells [39]. Although structurally and functionally different, the VacA and GGT proteins might share a common feature - the elevation of intracellular cAMP levels. Indeed, previous work has suggested that the GGT protein modulates intracellular cAMP [17]. In accordance, we observed that the VacA and GGT mutants did not induce an increase in intracellular cAMP. Our results support the notion that any cAMP inducer can activate Foxp3 and miR-155 expression. These previous observations and our new findings lead to a model that connects *H. pylori* LPS and the secreted factors VacA and GGT with an increase in intracellular cAMP and expression of the Foxp3-miR-155 duet.

The main consequence of miRNA expression is the post-transcriptional silencing of targeted genes. Many targets of miR-155 have previously been described (reviewed in [40]). Bioinformatic predictions designated PKIα as a putative target of miR-155. This protein acts as a pseudo-substrate for PKA to inhibit PKA catalytic subunits [41]. We propose the scenario in which miR-155 targets PKIα to prevent the inhibition of PKA, thereby maintaining a positive feedback loop of its own expression. An exhaustive list (∼100) of miR-155 targets has recently been published [42]; one of the published targets, the phosphodiesterase PDE3A, is involved in the turnover of cAMP. Besides miR-155, Foxp3 was also shown to support increasing levels of cAMP in immune cells [38]. Interestingly, another study suggested that PDE3B is one of the most Foxp3-repressed genes in regulatory T cells [43]. Foxp3 affects the response of regulatory T cells to environmental stimuli by modulating their cell surface and signalling molecules. Adaptation results from the down-regulation of PDE3B to support normal homeostasis, metabolic functions and proliferation. In addition, previous workers have shown that Foxp3 represses, directly or indirectly, expression of another miRNA, miR-142-3p, which targets AC9 [44].^.^ Collectively, these findings draw attention to the importance of a functional cAMP pathway in Foxp3^+^ T cells.

Our study has revealed miR-155 as the only miRNA commonly regulated by *H. pylori* in different cell lineages (epithelial and hematopoietic), at least in the analysed timeframe. For the first time in the context of *H. pylori* infection, the mechanisms of miR-155 induction in T cells have been dissected. Bacterial miRNA inducers were identified (VacA, GGT and LPS) and shown to be activators of cAMP. In turn, cAMP was found to be necessary for Foxp3 induction. Finally, miR-155 was shown to repress the PKA inhibitor (PKIα) protein expression in order to facilitate continuous intracellular cAMP production (see Figure S4). Taken together, these results establish a direct link between Foxp3 and miR-155 in human T cells and highlight the importance of cAMP in the miR-155 induction cascade elicited by *H. pylori* infection. We suggest the sequential induction of Foxp3 and miR-155 by *H. pylori* keeps the cAMP pathway functional in order to achieve a long-lasting modulation of the immune system [45].

## Materials and Methods

### Cell Culture and Reagents

Jurkat (ATCC, TIB-152), CCRF-CEM (ATCC, CCL-119), J774a (DSMZ, ACC-170), MKN74 (JCRB, JCRB-0255) and AGS (ATCC, CRL-1739) cell lines were maintained in culture in RPMI-1640 medium supplemented with 10% heat-inactivated FCS. HEK 293T cells (DSMZ, ACC-305) were maintained in DMEM medium supplemented with 10% heat-inactivated FCS and glutamine. For infection experiments, cells were starved of growth factors overnight. BMDM were isolated from C57Bl6 mice, as previously described [46]. Human peripheral blood monocytes (PBMCs) were isolated via the Ficoll-Hypaque density standard procedure. Forskolin (Serva, Germany), and PGE2 (Cayman Chemical, USA, Michigan) were used at final concentrations of 50 and 26 µM, respectively. The AC inhibitor MDL-12330A (Calbiochem, USA, New Jersey) was used at a final concentration of 50 µM. The monoclonal Foxp3 and anti-β actin antibodies were purchased from eBioscience (USA, San Diego) and Sigma (Germany, Steinheim), respectively. The monoclonal antibody anti-human CD4-FITC (clone MOPC-21) was obtained from BD Pharmingen (USA, San José). The isotype control was purchased from Becton Dickinson (USA, New Jersey). The monoclonal antibody anti-human Foxp3-PE (clone PCH101), the normal rat serum and the FoxP3 Staining Buffer Set were obtained from eBioscience (USA, San Diego). The PKIα antibody (SC-50349) was purchased from Santa Cruz Biotechnologies (USA, Santa Cruz) and used at a 1∶3000 dilution. LPS from *E.coli* (InvivoGen, USA, San Diego) was used at a final concentration of 100 ng/ml. The GGT inhibitor, acivicin (A2295), was purchased from Sigma (Germany, Steinheim) and used at a final concentration of 50 µM. The precursor of miR-155, miR-141 and the negative control (Pre-miR negative control 1) were purchased from Applied Biosystems (USA, Foster City).

### Bacterial Strains and Infection

*H. pylori* strains were grown on serum plates (GC agar plates containing 10 µg/l vancomycin, 5 µg/l trimethoprim and 2 µg/l nystatin) in a microaerobic atmosphere at 37°C. Wild-type strains used were P12, *H. pylori* 76 and SS1 (mouse adapted strains). The P12Δ*ggt* mutant was obtained by replacing the *ggt* gene (Hp1118) with a chloramphenicol resistance cassette. The double mutant P12Δ*vacA*Δ*ggt* was obtained by replacing the *vacA* mutant *ggt* gene by a kanamycin resistance cassette and grown on chloramphenicol and kanamycin plates. The mutants P12Δ*vacA*, P12Δ*ggt* were grown on plates supplemented with chloramphenicol. For all experiments, cells were infected with *H. pylori* for 3 h at an MOI of 100. Time course experiments covered 1, 3, 6, 9 and 12 h.

### Purification of Proteins and *H. pylori* LPS

The enzymes GGT, GGTm and the VacA protein were purified according to published protocols [17], [47]. VacA and both GGT proteins were added onto cell lines for 3 h at 10 µg/ml. GGT protein (1 µg) was added to human PBMCs. Before adding to cells, the VacA protein was activated by adding 10% (v/v) of 0.2N HCl [48], then neutralized by reaching a neutral pH. VacA and GGT preparations were tested negative for LPS contamination by using a limulus assay (LAL endosafe, Charles River, USA, Wilmington). LPS purification from *H. pylori* strain P12 was carried out as described previously [49] followed by DNase, RNase and proteinase K treatment. Quantification of the LPS fraction was carried out using a colorimetric based assay [50].

### Heat Inactivation of Bacteria and GGT

*H. pylori* was inactivated by incubation at 56°C for 45 min. Bacterial viability was then assessed by plating on GC agar plates and colony counting for 4 days at 37°C. GGT was inactivated by incubation at 95°C for 15 min.

### Microarray Analysis

Locked nucleic acids (LNA) modified microarray capture probes (miRCURY Probe Set, pre-release) were supplied at 480 pmol (Exiqon, Denmark, Vedbaek). Total RNA was isolated by the TRIzol Reagent RNA preparation method (Invitrogen, Germany, Karlsruhe). Quality control of the RNA preparations and quantification of total RNA amount was done using an Agilent 2100 bioanalyzer (Agilent Technologies, USA, Santa Clara) and a NanoDrop 1000 spectrophotometer. RNA samples were labeled with the miRCURY LNA microRNA Power Labeling Kit (Exiqon A/S) according the supplier’s instructions. Dual color hybridizations with 0.25–1 µg RNA per Hy-dye were performed for 16 h at 60°C with hybridization buffer (Exiqon, Denmark, Vedbaek) and LifterSlips (Erie Scientific, USA, Portsmouth). After hybridization and washing, arrays were scanned with a DNA Microarray Scanner BA (Agilent Technologies, USA, Santa Clara) at 5 µm resolution. Raw microarray image data were processed with the Image Analysis / Feature Extraction software G2567AA (Version A.8.5.1, Agilent). The extracted GEML files were loaded onto and analyzed with the Rosetta Resolver Biosoftware, Build 6.1 (Rosetta Biosoftware). A color-swap dye reversal experimental setting was applied. Ratio profiles comprising single hybridizations were combined in an error-weighted fashion to create ratio experiments. A twofold change expression cut-off for ratio experiments was applied together with anti-correlation of ratio profiles rendering the microarray analysis set highly significant (P-value<0.01), robust and reproducible. The data presented in this publication have been deposited in NCBIs Gene Expression Omnibus (GEO, http://www.ncbi.nlm.nih.gov/geo/) and are accessible through GEO Series accession number GSE18616.

### Total and Small RNA Isolation

Total RNA was harvested using TRIzol Reagent (Invitrogen, Germany, Karlsruhe). In PBMCs, RNA was isolated with the RNeasy kit (Invitrogen, Germany, Karlsruhe) and transcribed to cDNA. Small RNAs (<200 nt), were isolated after resuspension in TRIzol (Invitrogen, Germany, Karlsruhe) using the RNeasy MinElute Cleanup kit (Qiagen, Germany, Hilden). Total RNA from the antrum of human biopsies was isolated as described previously [19].

### qPCR

Quantitative RT-PCR for miR-155 was performed using TaqMan miRNA assays (Applied Biosystems, USA, Foster City). A RNU6B endogenous control was used for normalization. Following conversion of RNA (1 µg) to cDNA, gene expression levels were quantified using the ABI Prism 7900HT (Applied Biosystems, USA, Foster City). Relative expression was calculated using the comparative threshold cycle (Ct) method. Sybergreen-based real-time qPCR was performed for Foxp3, cytokeratin 18, CD3 and CD11c messenger RNA detection using 100 ng of total RNA and the following gene specific primers: Foxp3 RV (5′ATTGAGTGTCCGCTGCTTCT3′); Foxp3 FW (5′CAAGTTCCACAACATGCGAC3′); CK18 RV (5′ TGGCTTAATGCCTCAGAACTTTG3′); CK18 FW (5′ CCACCCGCCGGATAGTG3′); CD3ε RV (5′CCCAGTCCATCCCCAGAGA3′); CD3ε FW(5′CCCCATCCCAAAGTATTCCA3′); CD11c RV (5′TCCCTACGGGCCCCATAT3′); CD11c FW (5′GCCACCGCCATCCAAAA3′). Resulting data were normalized to β actin or GAPDH values using the following primers: β actin RV (5′CACCTTCACCGTTCCAGTTT3′); β actin FW (5′GATGAGATTGGCATGGCTTT3′); GAPDH RV (5′ATGCCAGTGAGCTTCCCGTTCAG3′) and GAPDH FW (5′GGTATCGTGGAAGGACTCATGAC3′).

### Generation of a Transient Foxp3 Knockdown

To silence Foxp3 expression by RNAi, CCRF-CEM cells were seeded into a 6-well plate and transfected with 100 nM siRNAs (Smart pool, Dharmacon, USA, Chicago) using HiPerFect transfection reagent (Qiagen, Germany, Hilden), according to the manufacturer’s instructions. Efficiency of gene silencing was validated by Western Blot analysis at 48 h post-transfection.

### miR-155 Over-Expression

T cells were seeded into a 6-well plate and transfected with 50 nM of miR-155 precursor or a control (Pre-miR control) (Ambion, USA, Austin) using HiPerFect transfection reagent (Qiagen, Germany, Hilden) following the manufacturer’s instructions. At 24 h post-transfection, over-expression was checked by qPCR.

### Luciferase Assay

A PCR fragment containing the target region of the 3′ÚTR of PKIα was ligated to the firefly luciferase plasmid pGL3-5 (a modified pGL3 (Promega, USA, Madison) with a different multiple cloning site). The resulting plasmid was named PKIa UTR. The complementary sequence of hsa-miR-155 was ligated to the pGL3-5 plasmid to obtain the ‘miR-155 indicator plasmid’.

HEK 293T cells were grown in 96-well plates and transfected with 200 nM of miR-155 precursor (Pre-155), miR-141 precursor (Pre-141) or the negative control (Pre-NC). Six hours later, the cells were co-transfected using Lipofectamine 2000 (Invitrogen, Germany, Karlsruhe) with 22.5 ng of *Renilla* luciferase plasmid (pRL-SV40, Promega, USA, Madison) and either 225 ng of PKIα UTR or the miR-155 indicator plasmid. Luciferase activities were determined 16 h post-transfection using the Dual- Glo Luciferase Reporter Assay (Promega, USA, Madison).

### Intracellular cAMP Measurement

T cells were infected with *H. pylori* P12 and the single mutant lacking the *vacA* and *ggt* genes. Forskolin was used as a positive control. Thirty minutes post-infection, cells were collected; lysed and intracellular cAMP levels were measured using the cAMP Parameter EIA kit (R&D Systems, USA, Minneapolis) according to the manufacturer's instructions.

### Western Blot

CCRF-CEM cells were lysed in Laemmli buffer. Cell lysates (25 µg) were loaded on SDS PAGE 10% gels and proteins transferred to PVDF membrane and blocked in TBS buffer containing 1% Tween 20 and 3% (W/V) BSA. The anti-Foxp3 antibody was used at a 1∶500 dilution; the anti-PKIα and the anti-β actin antibodies were used at a 1∶3000 dilution. Blots were quantified using ImageJ software.

### FACS Analysis

After infection, Jurkat cells were washed with PBS (supplemented with 0.5%BSA/0.02%NaN_3_) and stained with an anti-human CD4-FITC antibody. The intracellular staining of Foxp3 with the anti-FoxP3-PE antibody was carried out using the Foxp3 Staining Buffer Set (eBioscience). Ten thousand cells per sample were analysed in a FACSCalibur flow cytometer using CellQuest software (BD Biosciences, USA, San José).

### WST1 Proliferation Assay

CCRF-CEM cells were left untreated or incubated with 50 µM acivicin for 4 h. 1% Triton X-100 was added to untreated cells as a negative control for viability. The WST1 assay was performed according to the manufacturer’s instructions (Roche).

### CagA Phosphorylation Assay

Gastric epithelial cells (AGS) were left non-infected or infected for 3 h with *H. pylori* strain P12, the mutant P12Δ*cagPAI* and the mouse adapted strain Hp76. Cell lysates were obtained in 1x Laemmli buffer (60 mM Tris, pH 6.8, 2% SDS, 10% glycerol, 0.1% bromophenol blue) and analyzed by Western blotting with *H. pylori* antibody (Biomol, 1∶3000), CagA antibody (Santa Cruz, 1∶1000), phospho-Tyr (PY99) antibody (Santa Cruz, 1∶1000) and α-tubulin as a loading control (Sigma, 1∶1000). The P12Δ*cagPAI* mutant was used as a negative control of CagA phosphorylation.

### Statistical Analysis

All statistical tests were carried out using one-way ANOVA, followed by an unpaired Student's t-tests using Excel (Microsoft). Significance was set at p≤0.05.

## Supporting Information

Figure S1 Quantification of different cellular types in human gastric biopsies upon *H. pylori* infection. Human biopsies from the antrum of four different patients (13 to 77) were analyzed by real time RT-PCR before (day 0) and upon 80 days (day 80) of *H. pylori* infection. The experiment was carried out using cell markers such as cytokeratin 18 for epithelial cells, CD3 for T cells and CD11c specific of human macrophages and dendritic cells. Results showed a slight decrease of epithelial cells, a stabilization of T cell numbers but a very strong recruitment of macrophages and/or dendritic cells ranging from 4 to 16 fold.
(0.13 MB TIF)

Figure S2 cagPAI is not functional in Hp76 strain. CagA phosphorylation was checked in AGS gastric epithelial cells upon 3 hours of infection with strains P12 and Hp76. P12DcagPAI was used as a negative control. Lysates of non-infected (NI) and infected cells were harvested in Laemmli buffer and probed with *H. pylori*, cagA, phospho-cagA (p-CagA) and tubulin antibodies. Western blots show that upon infection with Hp76 strain or the P12DcagPAI mutant, no cagA phosphorylation is observed.
(0.43 MB TIF)

Figure S3 WST1 viability assay upon acivicin treatment. CCRF-CEM cells were untreated (NI) or incubated with 50 µM of acivicin for 3 hours. Cells were also treated with 1% Triton X-100 as a negative control of cellular viability. Results show that acivicin treatment does not result in significant loss of cell proliferation.
(0.11 MB TIF)

Figure S4 Schematic representation of miR-155 induction by *H. pylori* in human T cells. Bacterial factors including the LPS and the secreted VacA (vacuolating toxin A) and GGT (γ-glutamyl transpeptidase) induce intracellular cAMP (cyclic adenosine monophosphate) elevation via non-identified mechanisms. Through a previously well-established cascade involving the PKA (protein kinase A) and the transcription factor CREB (cAMP responsive element-binding), cAMP elevation triggers Foxp3 (Forkhead box p3) and Cox2 (Cycoloxygenase 2) expression. On one side, cox2 will allow PGE2 (Prostaglandin E2) formation, which will then be secreted in order to bind to its EP2 membrane receptor, activate the Gαs protein which in turn activates adenylate cyclase and hence increase cAMP levels. On the other side, the Foxp3 protein will enable the formation of the BIC transcript. Cytoplasmic export and maturation processes of this transcript will result in miR-155 formation. miR-155 repression of PKIα (protein kinase α inhibitor), a natural inhibitor of PKA then facilitates intracellular cAMP production.
(1.54 MB EPS)
