# Supplementary figures and images for: Helicobacter pylori Induces miR-155 in T Cells in a cAMP-Foxp3-Dependent Manner
Source: PLoS One. 2010 Mar 2;5(3):e9500. doi: 10.1371/journal.pone.0009500 (PMC2830477; doi:10.1371/journal.pone.0009500)

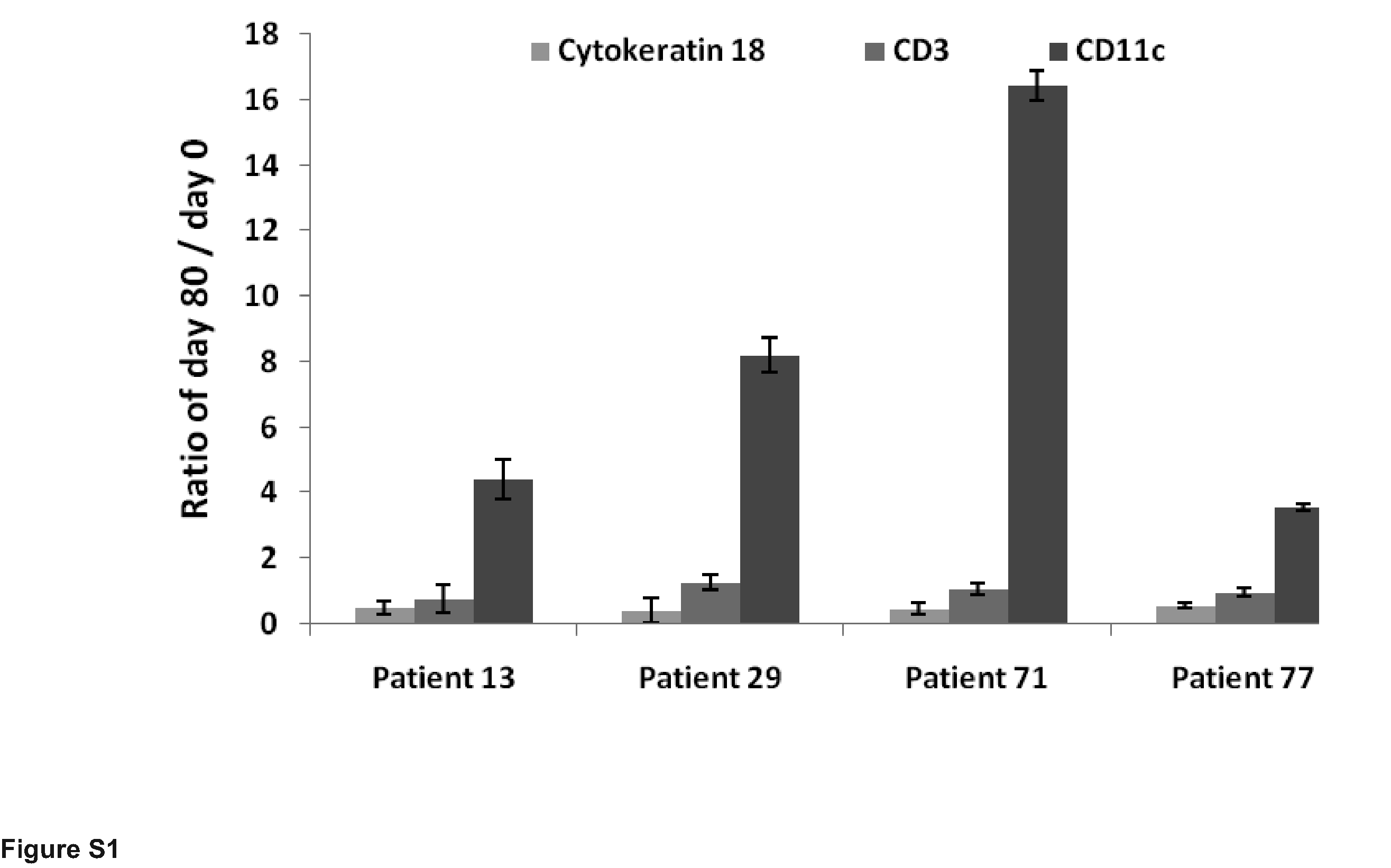

Supplement: Figure S1 — Quantification of different cellular types in human gastric biopsies upon H. pylori infection. Human biopsies from the antrum of four different patients (13 to 77) were analyzed by real time RT-PCR before (day 0) and upon 80 days (day 80) of H. pylori infection. The experiment was carried out using cell markers such as cytokeratin 18 for epithelial cells, CD3 for T cells and CD11c specific of human macrophages and dendritic cells. Results showed a slight decrease of epithelial cells, a stabilization of T cell numbers but a very strong recruitment of macrophages and/or dendritic cells ranging from 4 to 16 fold. (0.13 MB TIF) [file pone.0009500.s001.tif]

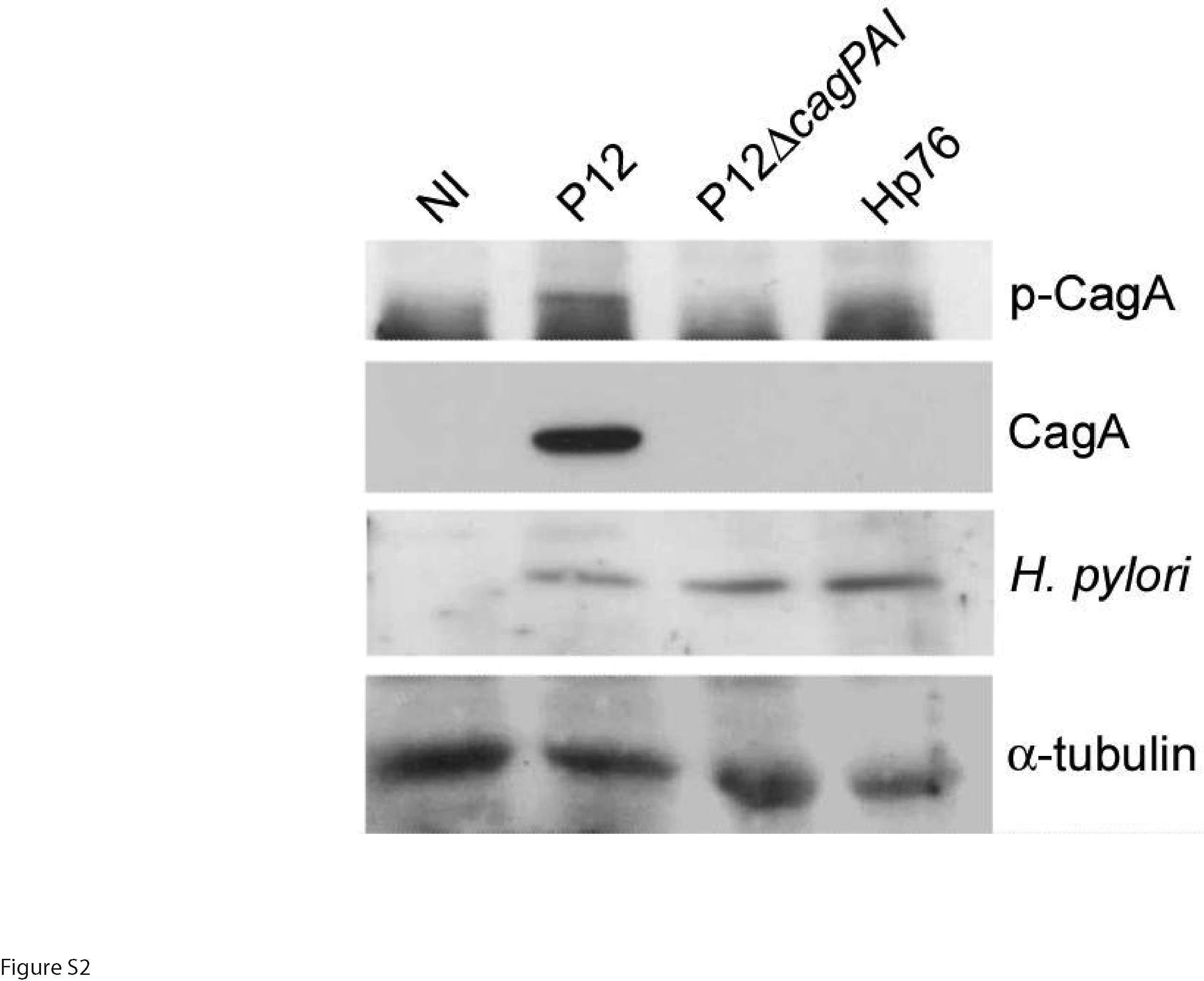

Supplement: Figure S2 — cagPAI is not functional in Hp76 strain. CagA phosphorylation was checked in AGS gastric epithelial cells upon 3 hours of infection with strains P12 and Hp76. P12DcagPAI was used as a negative control. Lysates of non-infected (NI) and infected cells were harvested in Laemmli buffer and probed with H. pylori, cagA, phospho-cagA (p-CagA) and tubulin antibodies. Western blots show that upon infection with Hp76 strain or the P12DcagPAI mutant, no cagA phosphorylation is observed. (0.43 MB TIF) [file pone.0009500.s002.tif]

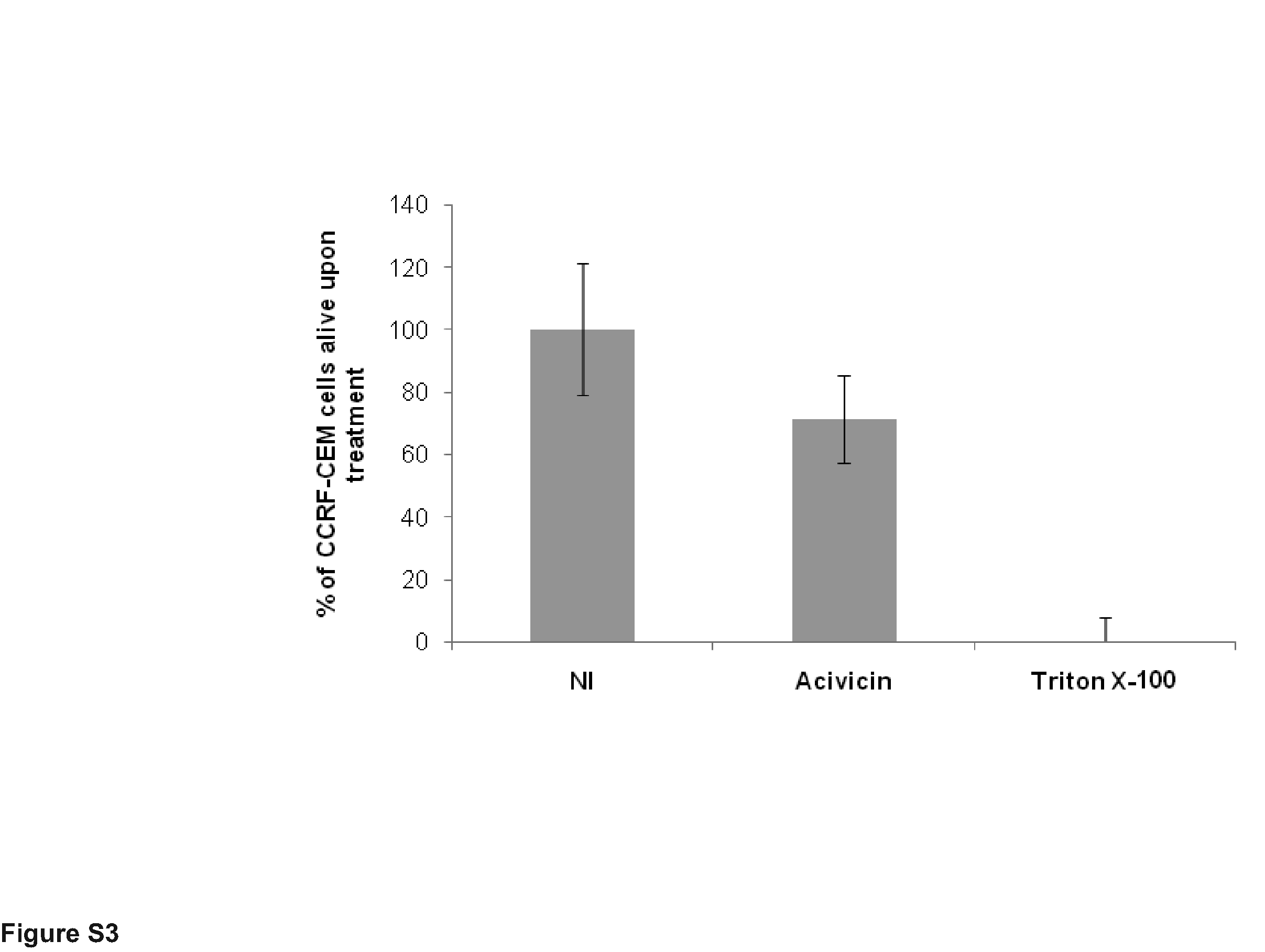

Supplement: Figure S3 — WST1 viability assay upon acivicin treatment. CCRF-CEM cells were untreated (NI) or incubated with 50 µM of acivicin for 3 hours. Cells were also treated with 1% Triton X-100 as a negative control of cellular viability. Results show that acivicin treatment does not result in significant loss of cell proliferation. (0.11 MB TIF) [file pone.0009500.s003.tif]
